# Alterations in learning-related cortical activation and functional connectivity by high-definition transcranial direct current stimulation after stroke: an fNIRS study

**DOI:** 10.3389/fnins.2023.1189420

**Published:** 2023-06-02

**Authors:** Heegoo Kim, Gihyoun Lee, Jungsoo Lee, Yun-Hee Kim

**Affiliations:** ^1^Department of Physical and Rehabilitation Medicine, Center for Prevention and Rehabilitation, Heart Vascular Stroke Institute, Samsung Medical Center, Seoul, Republic of Korea; ^2^Department of Health Sciences and Technology, Samsung Advanced Institute for Health Science & Technology (SAIHST), Sungkyunkwan University, Seoul, Republic of Korea; ^3^Department of Rehabilitation Medicine, CHA Bundang Medical Center, CHA University, Seongnam, Republic of Korea; ^4^Department of Physical and Rehabilitation Medicine, Sungkyunkwan University School of Medicine, Suwon, Republic of Korea; ^5^Department of Medical IT Convergence Engineering, Kumoh National Institute of Technology, Gumi, Republic of Korea; ^6^Haeundae Sharing and Happiness Hospital, Pusan, Republic of Korea

**Keywords:** high-definition transcranial direct stimulation, functional near-infrared spectroscopy, stroke, motor learning, cortical activation, functional connectivity

## Abstract

**Introduction:**

Motor learning is a key component of stroke neurorehabilitation. High-definition transcranial direct current stimulation (HD-tDCS) was recently developed as a tDCS technique that increases the accuracy of current delivery to the brain using arrays of small electrodes. The purpose of this study was to investigate whether HD-tDCS alters learning-related cortical activation and functional connectivity in stroke patients using functional near-infrared spectroscopy (fNIRS).

**Methods:**

Using a sham-controlled crossover study design, 16 chronic stroke patients were randomly assigned to one of two intervention conditions. Both groups performed the sequential finger tapping task (SFTT) on five consecutive days, either with (a) real HD-tDCS or (b) with sham HD-tDCS. HD-tDCS (1 mA for 20 min, 4 × 1) was administered to C3 or C4 (according to lesion side). fNIRS signals were measured during the SFTT with the affected hand before (baseline) and after each intervention using fNIRS measurement system. Cortical activation and functional connectivity of NIRS signals were analyzed using a statistical parametric mapping open-source software package (NIRS-SPM), *OptoNet* II^®^.

**Results:**

In the real HD-tDCS condition, oxyHb concentration increased significantly in the ipsilesional primary motor cortex (M1). Connectivity between the ipsilesional M1 and the premotor cortex (PM) was noticeably strengthened after real HD-tDCS compared with baseline. Motor performance also significantly improved, as shown in response time during the SFTT. In the sham HD-tDCS condition, functional connectivity between contralesional M1 and sensory cortex was enhanced compared with baseline. There was tendency toward improvement in SFTT response time, but without significance.

**Discussion:**

The results of this study indicated that HD-tDCS could modulate learning-related cortical activity and functional connectivity within motor networks to enhance motor learning performance. HD-tDCS can be used as an additional tool for enhancing motor learning during hand rehabilitation for chronic stroke patients.

## 1. Introduction

Stroke is a leading cause of disability, and many stroke patients live with lasting sensorimotor impairment (Anwer et al., 2022). Long-term disability in upper extremity motor function due to stroke can cause major challenges in performing activities of daily living (Langhorne et al., 2009), social participation (Sveen et al., 1999), and returning to work (Baldwin and Butler, 2006). Understanding the changes that occur in motor-related neurological mechanisms after stroke might facilitate the development of appropriate therapies that could enable better functional improvement.

Relearning specific motor skills required to complete daily tasks is a key component of stroke rehabilitation for upper extremity motor function. Learning a new motor skill requires the operation of several distinct motor learning processes that rely on different neuronal substrates (Spampinato and Celnik, 2021). On the cortical level, the prefrontal cortices and parietal lobes, which comprise the frontoparietal network, are engaged both in forming motor memory in the early learning phase and in delayed recall of learned motor skills (Doyon et al., 2003; Lewis and Miall, 2003). The motor cortex, including the primary motor cortex (M1), premotor cortex (PM), and supplementary motor area (SMA), is strongly interconnected with the frontoparietal network at the cortical level (Dahms et al., 2020). Also, for appropriate motor output of learned skills to the descending motor system, motor cortices must interact with the striatum and other parts of the basal ganglia (BG) (Dahms et al., 2020). After stroke, activity-dependent adaptations within the distributed neural networks can be induced by practicing skilled movements and changes in cortical representations (Kami et al., 1995; Karni et al., 1998), despite specific lesions. However, it is difficult to draw clear conclusions about the neural mechanisms used to recruit brain areas during motor learning because of the heterogeneity of stroke.

Transcranial direct current stimulation (tDCS) techniques have been used to alter neuronal activity and establish causal relationships between motor network components and behavioral outcomes to improve motor learning (Ammann et al., 2016) by controlling the polarity of induced electrical stimulation (Dissanayaka et al., 2017). Recently, high-definition tDCS (HD-tDCS) has been developed to increase the spatial precision of current delivery to a targeted cortical region using arrays of small electrodes (Villamar et al., 2013). A 4 × 1 ring configuration is one common arrangement of HD-tDCS electrodes to concentrate peak stimulation in a target region (Lefebvre et al., 2019). A previous brain modeling study that used high-resolution magnetic resonance imaging (MRI) demonstrated that the area of cortex undergoing modulation using a 4 × 1 ring configuration for HD-tDCS is more highly focused than that with the bipolar montage used in conventional tDCS (Datta et al., 2009). As measured by behavioral and neurophysiological parameters, HD-tDCS has been shown to improve motor learning capacity (Iannone et al., 2022) and have long-lasting effects in enhancing motor cortex excitability (Kuo et al., 2013). Taken together, the results of previous research indicate a need to clarify the neuronal mechanisms that underlie the modulatory effects of HD-tDCS.

Neuroimaging techniques are used to expand understanding of neuronal mechanisms (Esmaeilpour et al., 2020). Functional near-infrared spectroscopy (fNIRS) is a noninvasive optical imaging technique that can illustrate cortical activity by quantifying the concentrations of oxyhemoglobin (oxyHb) and deoxyhemoglobin (deoxyHb) using continuous-wave light (650–950 nm) emitted through the skull into the brain (Ferrari and Quaresima, 2012). Unlike conventional functional neuroimaging modalities, such as functional MRI (fMRI) and positron emission tomography (PET) (Leff et al., 2011; Ferrari and Quaresima, 2012), fNIRS has a relatively high tolerance to motion artifacts as it continuously detects hemodynamic responses, even during motor tasks. Therefore, the use of fNIRS in clinical trials is expanding (Delorme et al., 2019; Lee S.-H. et al., 2020; Huo et al., 2021). A recent fNIRS study suggested that the resting-state functional connectivity of the dorsolateral prefrontal cortex increased after HD-tDCS in healthy subjects (Yaqub et al., 2018). An fNIRS study in stroke patients demonstrated that HD-tDCS could rebalance interhemispheric cortical activity and reduce the hemodynamic burden in the affected hemisphere during simple finger tapping tasks (Kim et al., 2022). Furthermore, the usefulness of an fNIRS study on the effect of focal HD-tDCS stimulation on upper limb motor function in stroke patients was proposed (Muller et al., 2021). However, whether HD-tDCS modulates both cortical activation and functional connectivity during motor learning after stroke remains unclear.

The purpose of this study was to investigate the changes of cortical activation and functional connectivity during motor learning task with affected hand in stroke patients. We hypothesized the cortical activation and functional connectivity would show different patterns depending on application of HD-tDCS on ipsilesional M1 in stroke patients. In this study, we used fNIRS to investigate how HD-tDCS to ipsilesional motor areas of stroke patients affected cortical activation during motor learning with the affected hand compared with sham HD-tDCS. We also examined how HD-tDCS application induces changes in functional connectivity of ipsilesional and contralesional M1 during motor learning with the affected hand in stroke patients.

## 2. Methods

### 2.1. Participants

Potential participants were recruited from an outpatient stroke rehabilitation clinic at Samsung Medical Center in Seoul, Republic of Korea, from June 2021 to June 2022. Clinicians in rehabilitation medicine identified suitable participants who meet the inclusion and exclusion criteria for this study and obtained informed consent from those subjects. Twenty-one chronic stroke patients enrolled in this study. Among them, five patients withdrew consent before intervention for personal reasons, thus, 16 patients (7 males and 9 females, mean age 56.8 ± 13.0 years) completed the study protocol. The inclusion criteria were unilateral hemiparetic stroke (both ischemic and hemorrhagic), age between 19 and 80 years, chronic stroke symptoms for more than 6 months, lesions including BG, and the ability to move individual fingers. The exclusion criteria were a history of psychiatric disease, significant neurological disease other than stroke, metal implants, and contraindications to tDCS application (Russo et al., 2017). Written informed consent was provided by all patients before participation. The patient demographics are described in Table 1, and the lesion map is presented in Supplementary Figure 1. The lesions were manually drawn on T1-weighted structural MRI with lesion mapping software (MRIcro Software).^1^ The lesions were normalized to the standard Montreal Neurological Institute (MNI) space and overlaid on a template of the MNI space. For patients with lesions on the right side, the lesions were flipped to the left side to better visualize the distribution. The experimental procedures were approved by the Ethics Committee of Samsung Medical Center. This study was registered at ClinicalTrials.gov (NCT04903457).

**Table 1:** Demographic information of participants.

Subject number	Sex	Age (years)	Onset duration (months)	Side of lesion	Location of lesion	Type of stroke	Allocated condition order	Handedness
1	M	58	64.7	Rt.	BG, CR	Infarction	Condition 2—condition 1	Rt. handed
2	M	38	51.7	Lt.	BG, CR	Infarction	Condition 1—condition 2	Rt. handed
3	F	69	8.7	Lt.	BG, CR	Infarction	Condition 1—condition 2	Bi-handed
4	F	64	31.2	Lt.	BG, CR	Infarction	Condition 1—condition 2	Rt. handed
5	F	54	15.6	Rt.	BG, CR	Infarction	Condition 2—condition 1	Rt. handed
6	F	76	53.5	Rt.	BG, CR	Infarction	Condition 2—condition 1	Rt. handed
7	M	48	67.9	Lt.	BG	Hemorrhage	Condition 2—condition 1	Rt. handed
8	M	33	67.4	Lt.	BG, CR	Hemorrhage	Condition 1—condition 2	Rt. handed
9	F	55	122.0	Lt.	BG, CR	Infarction	Condition 2—condition 1	Rt. handed
10	F	71	175.5	Lt.	BG, CR	Hemorrhage	Condition 1—condition 2	Rt. handed
11	M	58	73.2	Rt.	BG, CR	Infarction	Condition 2—condition 1	Rt. handed
12	F	37	45.6	Lt.	BG, CR, TH	Hemorrhage	Condition 2—condition 1	Rt. handed
13	F	65	130.8	Lt.	BG, CR	Hemorrhage	Condition 2—condition 1	Rt. handed
14	F	62	197.0	Rt.	BG, CR	Infarction	Condition 1—condition 2	Rt. handed
15	M	71	68.0	Rt.	BG, CR	Infarction	Condition 1—condition 2	Rt. handed
16	M	50	38.4	Lt.	BG, CR	Hemorrhage	condition 1 – condition 2	Rt. handed

M, male; F, female; Rt., right; Lt. left; BG, basal ganglia; CR, corona radiata; TH, thalamus.

### 2.2. Study design

**Figure 1:**
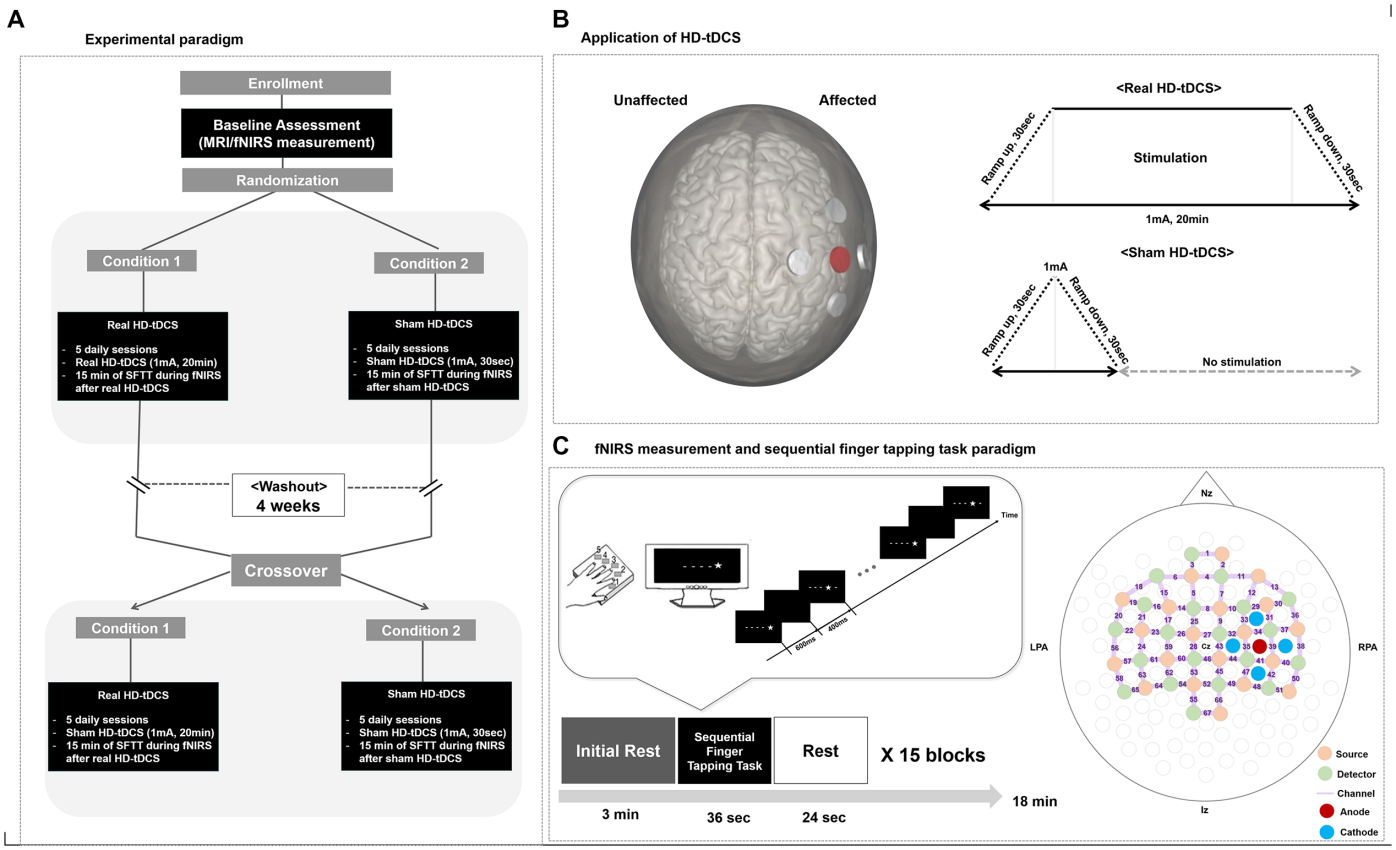
Study design. **(A)** Experimental paradigm. **(B)** Application of HD-tDCS. When a participant’s lesion was on the right side, the anode was placed on C4, and the cathodes were placed on C2, C6, FC4, and CP3. When a participant’s lesion was on the left side, the anode was placed on C3, and the cathodes were placed on C1, C5, FC3, and CP5. In the real HD-tDCS condition, a constant current was delivered at 1 mA for 20 min, with ramp-up and-down phases of 30 s. In the sham HD-tDCS condition, current was ramped up from 0 to 1 mA during the first 30 s, then ramp-down to 0 mA during the next 30 s, and remain at 0 mA for the next 20 min. **(C)** fNIRS measurement during the SFTT and the fNIRS topomap. A star appeared on the black screen for 600 ms, and then an empty black screen appeared for 400 ms after the star disappeared. HD-tDCS, high-definition transcranial direct current stimulation; fNIRS, functional near-infrared spectroscopy; SFTT, sequential finger tapping task; Nz, nasion; Iz, inion; LPA, left pre-auricular; RPA, right pre-auricular.

Using a sham-controlled, double-blind, crossover study design, all participants completed 10 days of HD-tDCS intervention. Before the intervention, all participants underwent MRI to examine lesion location and volume. At the same visit, fNIRS measurements were conducted during 15 min of the sequential finger tapping task (SFTT) to assess the initial motor learning capacity of each participant. Referring to the experimental protocols of previous tDCS cross-over studies (Gãlvez et al., 2013; Hamoudi et al., 2018), each participant underwent treatment with 2 HD-tDCS conditions for 5 consecutive days (days 1 to 5), separated by a 4-week washout period, in random order of intervention: (a) condition 1: 20 min of real HD-tDCS stimulation (real HD-tDCS) over the affected motor area and (b) condition 2: sham stimulation that applied the current used in the actual stimulation only during the 30-s ramp-up and-down periods (sham HD-tDCS). If a patient was first allocated to condition 1, that patient underwent the condition 2 process after the 4-week washout window period. The order of these treatments was randomly allocated. To measure hemodynamic changes during a motor learning task, fNIRS was conducted during a 15-min of SFTT after HD-tDCS application on every intervention day. In addition, to examine motor performance, each participant’s accuracy and response time during the SFTT were measured along with fNIRS measurements. The study design is illustrated in Figure 1A.

### 2.3. High-definition tDCS

A battery-driven Starstim 8 tDCS system (Neuroelectrics^®^, Barcelona, Spain) was used in a 4 × 1 ring configuration of HD electrodes (surface: 3.14 cm^2^; current density, anode, 0.32 mA/cm^2^; each cathode, ~0.08 mA/cm^2^) to deliver a constant direct current to the affected hemisphere. The anode, which was the center electrode of the 4 × 1 ring montage of HD electrodes, was placed on the scalp overlying C3 or C4 (based on the 10–20 system) to cover the ipsilesional motor cortical area. The four cathodes surrounded the anode at a center-to-center distance of 3.5 cm. Thus, when a participant’s lesion was on the right side, the anode was placed on C4, and the cathodes were placed on C2, C6, FC4, and CP4. When a participant’s lesion was on the left side, the anode was placed on C3, and the cathodes were placed on C1, C5, FC3, and CP3. A constant current of 1 mA was delivered for 20 min, with 30-s ramp-up and-down phases. In the sham procedure, stimulation was applied to the same area in the same electrode montage, but real stimulation was provided only during the ramp-up and-down periods to provide the same skin tingling sensation (Martïnez-Përez et al., 2020). First, a period of “ramping up” is administered, in which the stimulation reaches the maximum programmed current (e.g., 30 s to reach 1 mA). Ramping up is then followed by a short stimulatory period, in which the participant receives stimulation for a few seconds. Finally, “ramping down” involves the current gradually being switched off (Thair et al., 2017). HD-tDCS application is illustrated in Figure 1B.

### 2.4. Measurement of changes in hemodynamic response during the sequential finger tapping task

Changes in hemodynamic responses during the SFTT with the affected hand were measured in each patient on every intervention day. Using an fNIRS measurement system (NIRScout^®^; NIRx Medical Technologies, Berlin, Germany) on a multi-modal-compatible fNIRS platform, the hemodynamic response signals were obtained as optical changes collected in a continuous wave. The fNIRS system used two wavelengths, 760 and 850 nm, with a sampling rate of 10.25 Hz. With 20 sources and detectors, the fNIRS topomap consisted of 67 channels, with 3 cm between each source and detector. The fNIRS topomap was designed to cover nearly the whole brain area, including the frontal, motor, parietal, temporal, and occipital cortices (Figure 1C). During fNIRS measurements, all patients performed the SFTT with the affected hand. NIRStar 15.2 software (NIRx Medical Technologies) was used for signal acquisition, recording the raw fNIRS data, and obtaining signal quality indicators for measurement channels following hardware calibration. Channels with poor signal quality were identified using the following criteria and excluded from further analysis. First, channels with gain larger than 7, showing inadequate light detection, were rejected. The gain is calculated by the NIRx device during a calibration procedure performed prior to each experiment. In the NIRx system, gain values less than 7 are defined as optical signals within the range of 0.09–1.4 V and at noise levels less than 2.5% (Shoushtarian et al., 2020). If the acquired signal quality was poor during calibration, the contact between the scalp and analogous optodes was adjusted until the overall signal quality was acceptable.

An SFTT protocol programmed using SuperLabPro^®^ 2.0 software (Cedrus, Co., Phoenix, AZ, United States) was used with all participants (Figure 1C). During the SFTT with fNIRS measurement, each patient was seated 50 cm from a computer monitor, and the affected hand performing the task was held in a supported position. As a visual cue on the monitor, a star appeared at any one of five positions arranged in a horizontal line on the computer screen in front of the participant. The participant was instructed to use their affected fingers to press the button on a customized keyboard that corresponded to the stimulus presented on the screen as quickly and accurately as possible (thumb = 1, index finger = 2, middle finger = 3, ring finger = 4, little finger = 5). The star appeared for 600 ms, after which the screen went blank for 400 ms. Each sequence was composed of 9 digits, and the task block included 15 repetitions of that sequence. Information about the sequence order was not provided to the participant for this implicit motor learning task. Three pre-determined sequences with the same difficulty were randomly assigned to the baseline, real HD-tDCS, and sham HD-tDCS conditions.

### 2.5. Measurement of motor performance during the sequential finger tapping task

Accuracy and response time during the SFTT were used to measure changes in motor performance of the affected hand at every intervention session. To measure SFTT performance, each patient’s mean response time and number of correct responses (accuracy) (Kim et al., 2006) were calculated with SuperLabPro^®^ software. The response time was defined as the mean time required for the patient to press the correct key after appearance of the stimulus on the screen. The accuracy and response time were measured for 36 stimuli within each trial, with 15 trial blocks for each task. Also, we calculated the skill index (SI). Usually, when speed increases, accuracy decreases, and vice versa. The SI is used to compensate for the trade-off between speed and accuracy (Cuypers et al., 2013). In other words, the SI considers both the accuracy and response time parameters during the task and was calculated using the following formula.


$$\mathrm{SI}=\frac{\mathrm{Percentage of correct responses}\;\left(\%\right)}{\mathrm{Mean response time}\;\mathrm{per}\;\mathrm{block}\;\left(\mathrm{msec}\right)}$$

### 2.6. fNIRS data analysis

The fNIRS data for patients with a left-side lesion were flipped to the fNIRS channels on the opposite side, so that the lesion location for all subjects could be analyzed on the same side. The cortical activation map produced during the SFTT with the affected hand was analyzed using statistical parametric mapping (SPM) analysis with the Near-Infrared Spectroscopy-Statistical Parametric Mapping open-source software package (NIRS-SPM)^2^ (Tak et al., 2008) implemented in MATLAB^®^ (MathWorks, Inc., Natick, MA, United States). To test for significant changes in oxyHb concentration during task blocks compared with rest blocks, a general linear model was used with a canonical hemodynamic response curve (Ye et al., 2009). Then, the statistical contrast in reference to the base signal was tested, and cortical activity was presented as the *t*-value during experiment. In group analysis of all subjects, statistical analysis was performed based on the individual-level beta values to determine the activated channels. Then, the *t*-statistic maps computed for group analysis were plotted onto a conventional brain template aligned to the affected hemisphere, and regions with significant differences in oxyHb concentration were identified (*p* < 0.05, uncorrected) (Benjamini and Hochberg, 1995). Individual-level *t*-values for all 67 channels were extracted to statistically analyze the *t*-value for each channel. Then, the *t*-values of each channel were presented as individual regions of interest (ROIs) that were selected by fNIRS optode location decider (fOLD) channels (Zimeo Morais et al., 2018) in MATLAB^®^.

The analysis of functional connectivity between the bilateral M1 and other cortical regions using fNIRS data was performed using *OptoNet* II^®^ software (25 March 2021),^3^ which is a MATLAB-based application for functional cortical connectivity analysis of fNIRS signals (Lee et al., 2019; Lee G. et al., 2020). The functional connectivity between the bilateral M1 and other cortical regions was estimated by analyzing the phase-locking value (PLV) in *OptoNet* II^®^. The PLV can indicate synchrony between two recording sites in a precise frequency range and uses responses to repeated stimuli to search for latencies at which the phase difference between signals varies minimally across trials (phase-locking) (Lachaux et al., 1999). The intertrial variability of this phase difference was measured using the PLV; if the phase difference varied minimally across trials, the PLV was close to 1; otherwise, it was close to zero (Lachaux et al., 1999). After extracting the PLV from each of the 15 task blocks in each SFTT trial for each individual, the PLVs for each block were averaged. Because fNIRS channels for analyzing functional connectivity for cortical regions were determined by the fOLD channels (Zimeo Morais et al., 2018), they included the channels used to analyze *t*-values in SPM analysis as follows: medial pre-frontal (MPF), Ch. 1, 2, 3, 4; ipsilesional frontal area (Fr_Ipsi_), Ch. 10, 11, 12, 13, 29, 30; contralesional frontal area (Fr_Contra_), Ch. 6, 14, 15, 16, 18, 19; ipsilesional M1 (M1_Ipsi_), Ch. 34, 35, 39; contralesional M1 (M1_Contra_), Ch. 23, 24, 59; SMA, Ch. 9, 25, 27; ipsilesional PM (PM_Ipsi_), Ch. 31, 32, 33; contralesional PM (PM_Contra_), Ch. 17, 21, 26; ipsilesional sensory cortex (Sn_Ipsi_), Ch. 40, 41, 44; contralesional sensory cortex (Sn_Contra_), Ch. 57, 60, 61; ipsilesional parietal lobe (Pr_Ipsi_), Ch. 48, 49; contralesional parietal lobe (Pr_Contra_), Ch. 54, 64; ipsilesional temporal lobe (Tm_Ipsi_), Ch. 36, 38; contralesional temporal lobe (Tm_Contra_), Ch. 20, 56; and occipital lobe (Occ), Ch. 55, 66. The fNIRS signals were processed with normalization for each epoch to prevent signal distortion caused by differences between functional region groups in the number of fNIRS channels. The PLVs between the bilateral M1 and other ROIs were extracted to compare changes in functional connectivity at every measurement.

### 2.7. Statistical analysis

The data were analyzed using SPSS version 20 (SPSS, Inc., Chicago, IL, United States). To evaluate the normality of the distribution, the data were examined using the Kolmogorov–Smirnov test. The statistical significance of changes in the *t*-values of channels 35 and 59 and the PLVs from the fNIRS measurements was determined through three stages of analysis. First, repeated measures analysis of variance (RM-ANOVA) was used to confirm the interaction between conditions (real HD-tDCS and sham HD-tDCS) and changes in the *t*-values and PLVs of the fNIRS measurement on the five intervention days. Second, the Friedman test was used to examine the effects of days within each condition at each measurement because the *t*-values and PLVs were found to have non-parametric distributions. Third, the *t*-values and PLVs obtained for each intervention were compared with the baseline values and evaluated using the Wilcoxon signed-rank test. For statistical analysis of the SFTT variables, three stages of analysis were performed. First, RM-ANOVA was used to test the interaction between conditions and blocks of the SFTT for each measurement. Second, the Friedman test or RM-ANOVA, depending on the normality distribution of data, was used to assess the effects of the blocks within each condition on each intervention day. Third, SFTT variables in each block were compared with the first block on every measurement day using the Wilcoxon signed rank test. For all analyses, the level of significance was set at *p* = 0.05.

## 3. Results

### 3.1. Cortical activity analysis during the sequential finger tapping task using NIRS-SPM

**Figure 2:**
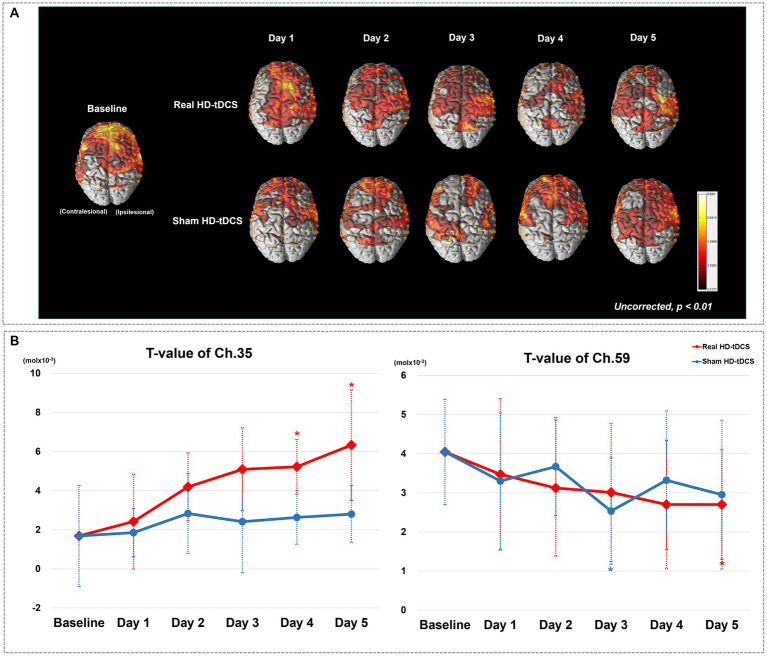
**(A)** Average cortical activation maps during the SFTT with the affected hand. SFTT, sequential finger tapping task. **(B)** *T*-value changes in the ipsilesional (channel 35) and contralesional (channel 59) M1 from baseline to day 5. Red and blue asterisks indicate statistical significance between baseline and each measurement day in the real HD-tDCS and sham HD-tDCS conditions, respectively (Wilcoxon signed rank test, *p* < 0.05).

Figure 2A shows changes in the average cortical activation in terms of oxyHb during the SFTT with the affected hand from baseline to day 5 in each condition, as illustrated by the NIRS-SPM analysis. On day 5 in the real HD-tDCS condition, the oxyHb concentration during SFTT with the affected hand increased primarily around the ipsilesional motor cortices. The changes in *t*-values for channel 35 and 59, which represent the ipsilesional and contralesional M1, respectively, are illustrated in Figure 2B. The *t*-value changes for those channels show no day × condition interaction. As the intervention progressed, the *t*-value of channel 35 increased from baseline to day 5 with statistical significance (Friedman test, ${Χ}^{2}$ = 16.828, df = 5, *p* = 0.005) in the real HD-tDCS condition. In the sham HD-tDCS condition, the *t*-value of channel 35 tended to increase as the intervention progressed, but the difference was not statistically significant. On days 4 and 5 in the real HD-tDCS condition, the increase in the *t*-value of channel 35 attained statistical significance compared to baseline (Wilcoxon signed-rank test, day 4, *p* = 0.034; day 5, *p* = 0.020). The *t*-value of channel 59 tended to decrease from baseline to day 5 without statistical significance in both the real HD-tDCS and sham HD-tDCS conditions. A significant decrease in channel 59 occurred on day 5 and day 3 in the real HD-tDCS and sham HD-tDCS conditions, respectively (Wilcoxon signed rank test, real HD-tDCS, *p* = 0.026; sham HD-tDCS, *p* = 0.008). The changes in the *t*-values of the ROI channels are presented in Table 2.

**Table 2:** Changes in *t*-values on cortical activation mapping through statistical parametric mapping at each intervention session.

			Real HD-tDCS					Sham HD-tDCS				
Region of interest	Channel	Baseline	Day 1	Day 2	Day 3	Day 4	Day 5	Day 1	Day 2	Day 3	Day 4	Day 5
MPF	Ch. 4	3.121 (3.959)	3.126 (5.304)	3.158 (4.484)	**2.699** ^*^ **(4.029)**	2.004 (4.195)	**1.672** ^*^ **(3.594)**	2.719 (2.601)	2.641 (3.831)	2.643 (3.646)	**2.145** ^*^ **(3.799)**	**2.546** ^*^ **(3.813)**
Fr_Ipsi_	Ch. 12	2.581 (2.532)	**1.357** ^*^ **(2.326)**	1.945 (2.447)	2.148 (3.429)	**1.961** ^*^ **(2.553)**	1.704 (2.658)	2.233 (2.396)	2.234 (3.910)	3.073 (1.751)	1.634 (2.919)	3.165 (2.630)
Fr_Contra_	Ch. 15	3.041 (3.517)	**1.671** ^*^ **(1.773)**	1.447 (2.532)	2.903 (4.609)	2.098 (2.945)	**0.672** ^*^ **(1.985)**	**1.876** ^*^ **(3.425)**	**2.499** ^*^ **(2.257)**	2.893 (3.827)	2.504 (1.419)	1.841 (2.849)
M1_Ipsi_	Ch. 35	2.512 (3.915)	2.354 (4.407)	2.882 (4.678)	3.662 (2.990)	**4.841** ^*^ **(4.092)**	**7.223** ^*^ **(5.944)**	1.163 (2.191)	1.850 (6.350)	2.315 (1.629)	1.656 (2.489)	1.886 (4.358)
M1_Contra_	Ch. 59	3.535 (3.527)	2.358 (5.889)	2.863 (2.279)	2.637 (4.802)	2.417 (3.296)	**1.692** ^*^ **(3.320)**	2.053 (3.534)	2.978 (4.052)	**2.004** ^**^ **(2.791)**	3.567 (4.156)	3.075 (5.753)
SMA	Ch. 27	2.321 (2.181)	**2.461** ^*^ **(3.226)**	1.697 (3.093)	3.248 (4.146)	0.530 (3.948)	1.493 (5.023)	0.975 (3.441)	1.647 (2.601)	−0.469 (4.862)	2.062 (4.559)	1.328 (4.650)
PM_Ipsi_	Ch. 32	1.543 (3.345)	3.131 (3.172)	2.361 (3.465)	3.375 (4.067)	2.911 (2.586)	2.813 (2.352)	1.804 (2.167)	1.461 (4.142)	2.166 (2.633)	1.092 (3.440)	0.915 (5.183)
PM_Contra_	Ch. 26	4.062 (3.566)	4.232 (4.801)	3.396 (3.223)	3.208 (5.522)	3.569 (4.354)	3.020 (3.563)	**2.021** ^*^ **(3.062)**	3.400 (4.046)	2.258 (2.796)	3.130 (3.479)	2.882 (6.079)
Sn_Ipsi_	Ch. 44	3.166 (3.354)	2.602 (4.356)	2.273 (6.092)	2.877 (3.850)	0.713 (3.432)	1.785 (4.842)	0.597 (2.484)	1.470 (2.347)	1.190 (1.996)	1.196 (2.701)	1.354 (3.683)
Sn_Contra_	Ch. 60	4.011 (3.488)	3.329 (5.164)	3.822 (3.172)	1.816 (5.338)	**1.601** ^*^ **(3.423)**	3.480 (3.407)	**1.794** ^*^ **(2.608)**	1.959 (4.117)	**2.510** ^*^ **(2.160)**	**2.587** ^*^ **(3.712)**	**3.083** ^*^ **(3.856)**
Pr_Ipsi_	Ch. 49	2.204 (3.050)	1.902 (5.931)	3.084 (2.755)	0.842 (3.336)	1.485 (3.021)	3.485 (3.466)	1.714 (2.185)	1.522 (2.746)	3.091 (4.339)	0.525 (2.980)	2.262 (4.712)
Pr_Contra_	Ch. 54	1.135 (2.386)	1.638 (4.267)	3.044 (4.554)	2.694 (4.367)	2.063 (4.323)	2.736 (2.608)	1.276 (2.930)	1.974 (4.650)	2.162 (3.349)	0.616 (6.269)	1.785 (4.218)
Tm_Ipsi_	Ch. 36	2.430 (3.173)	0.589 (1.841)	1.724 (2.782)	0.427 (3.520)	1.650 (2.667)	2.405 (2.469)	0.719 (1.802)	1.475 (4.526)	1.479 (2.969)	1.667 (2.624)	1.824 (1.521)
Tm_Contra_	Ch. 20	3.871 (3.695)	**2.461** ^*^ **(2.796)**	2.123 (4.231)	**1.183** ^*^ **(3.624)**	**0.976** ^*^ **(4.095)**	**1.053** ^*^ **(5.082)**	**1.608** ^*^ **(2.968)**	2.898 (3.806)	**1.473** ^*^ **(1.427)**	**0.814** ^*^ **(2.141)**	1.254 (2.935)
Occ	Ch. 66	1.975 (3.730)	2.590 (5.932)	2.424 (5.399)	1.699 (2.466)	1.995 (2.398)	2.951 (3.189)	1.592 (3.006)	2.068 (3.271)	2.732 (5.168)	1.580 (3.086)	1.646 (5.631)

All data are expressed as median (interquartile range). MPF, medial prefrontal cortex; Fr_Ipsi_, ipsilesional frontal area; Fr_Contra_, contralesional frontal area; M1_Ipsi_, ipsilesional primary motor cortex; M1_Contra_, contralesional primary motor cortex; SMA, supplementary motor area; PM_Ipsi_, ipsilesional premotor cortex; PM_Contra_, contralesional premotor cortex; Sn_Ipsi_, ipsilesional sensory cortex; Sn_Contra_, contralesional sensory cortex; Pr_Ipsi_, ipsilesional parietal cortex; Pr_Contra_, contralesional parietal cortex; Tm_Ipsi_, ipsilesional temporal lobe; Tm_Contra_, contralesional temporal lobe; Occ, occipital lobe. *Significant change compared with baseline (Wilcoxon-signed rank test, *p* < 0.05). Bold values mean significant change compared with baseline.

### 3.2. Functional connectivity analysis during sequential finger tapping task

**Figure 3:**
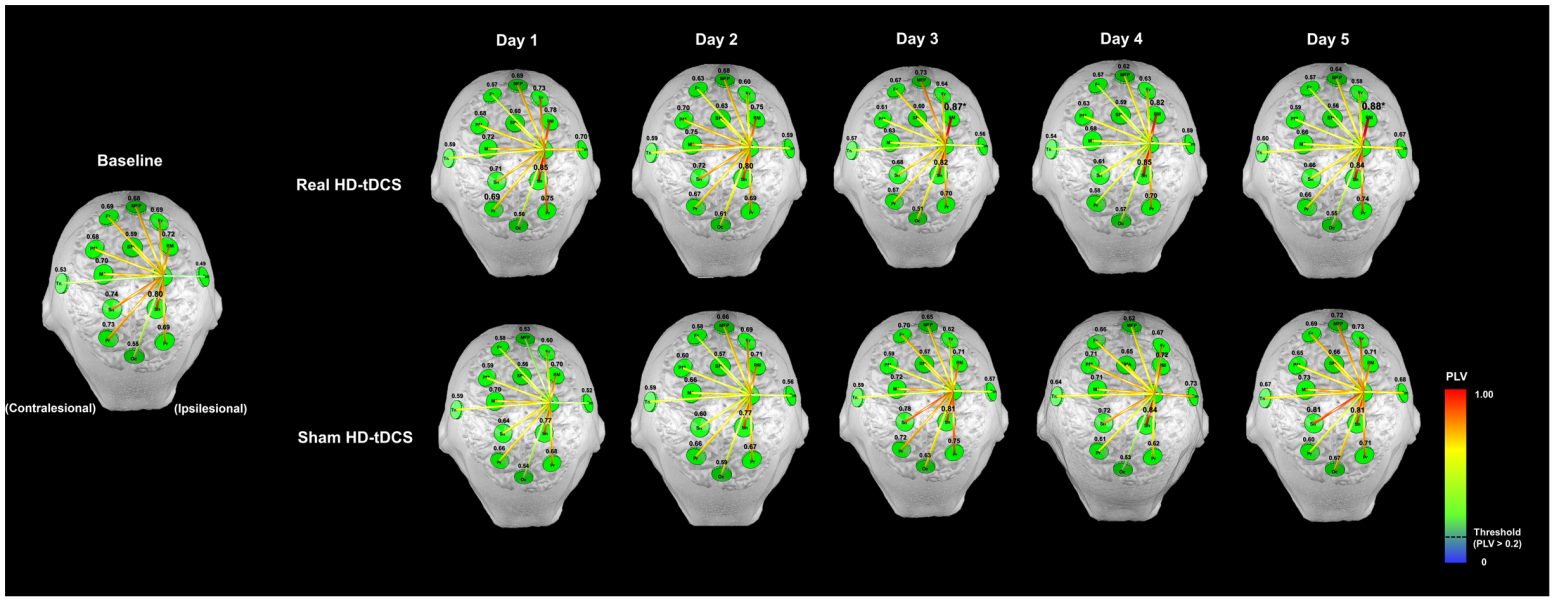
Changes in functional connectivity between the ipsilesional M1 and other ROIs during the SFTT with the affected hand. The letters in green circles indicate the names of the ROIs, and the colored lines represent functional connectivity between the ipsilesional M1 and each ROI. The numbers above each green circle are the mean PLV between the ipsilesional M1 and that ROI. The functional connectivity line is represented by a warmer color if the PLV was close to 1 and a cooler color if the PLV was close to 0, and only the high-value lines (threshold >0.2) are represented. The PLV between the ipsilesional M1 and PM increased on days 3 and 5 compared with baseline in the real HD-tDCS condition (Wilcoxon signed rank test, *p <* 0.05). MPF, medial prefrontal cortex; Fr, frontal area; M1, primary motor cortex; SMA, supplementary motor area; PM, premotor cortex; Sn, sensory cortex; Pr, parietal cortex; Tm, temporal lobe; Occ; occipital lobe.

Figure 3 shows changes in mean PLV between M1_Ipsi_ and the other ROIs during SFTT with the affected hand between baseline and the fifth day in each condition. The values above each ROI indicate the PLV between M1_Ipsi_ and each ROI in Figure 3. The changes in PLV at each ROI showed no day × condition interaction for any ROI. At baseline, the PLV between M1_Ipsi_ and Sn_Ipsi_ indicated a relatively strong connection compared with connections between M1_Ipsi_ and the other ROIs. From days 1 to 5, as the intervention progressed, the PLV between M1_Ipsi_ and PM_Ipsi_ showed a tendency to increase compared with baseline in the real HD-tDCS condition, but the difference was not statistically significant. The PLV between M1_Ipsi_ and Sn_Ipsi_ tended to increase as the days progressed in the real HD-tDCS condition but without statistical significance. The PLV between M1_Ipsi_ and PM_Ipsi_ increased from a baseline value of 0.70 ± 0.24 to 0.88 ± 0.08 (mean ± standard deviation) on day 3, which was a statistically significant change (Wilcoxon signed rank test, *p =* 0.007). On day 5, the PLV difference between M1_Ipsi_ and PM_Ipsi_ increased with statistical significance from baseline to 0.88 ± 0.08 (Wilcoxon signed-rank test, *p =* 0.017). The PLV between M1_Ipsi_ and Sn_Ipsi_ did not increase significantly in the real HD-tDCS condition compared with baseline on any day. In the sham HD-tDCS condition, the PLV between M1_Ipsi_ and other ROIs maintained a level similar to that at baseline. On day 5, relatively strong connections were shown between M1_Ipsi_ and Sn_Contra_ compared with baseline, but this was not statistically significant.

Changes in PLV at each ROI showed a day × condition interaction between M1_Contra_ and Fr_Ipsi_ was statistically significant (RM-ANOVA, *F* = 3.155, *p* = 0.015). The PLV between M1_Contra_ and Fr_Contra_ tended to decrease from baseline to day 5 in the real HD-tDCS condition. On the other hand, the PLV between M1_Contra_ and Fr_Contra_ increased significantly from baseline to day 5 in the sham HD-tDCS condition (Wilcoxon signed rank test, *p* = 0.039). The PLV between M1_Contra_ and Fr_Ipsi_ also tended to decrease without statistical significance from baseline to day 5 in the real HD-tDCS condition. However, the PLV between M1_Contra_ and Fr_Ipsi_ showed a tendency to increase in the sham HD-tDCS condition. The PLV between M1_Contra_ and Fr_Ipsi_ increased with significance on day 5 (Wilcoxon signed rank test, *p =* 0.020) in the sham HD-tDCS condition, and in the same condition, the PLV between M1_Contra_ and Sn_Contra_ increased significantly compared with baseline on days 3, 4, and 5 (Wilcoxon signed rank test, day 3, *p* = 0.011; day 4, *p* = 0.034; day 5, *p* = 0.023). Changes in the mean PLV between M1_Contra_ and the other ROIs during SFTT with the affected hand from baseline to the fifth day in each condition are presented in Supplementary Figure 2.

### 3.3. Statistical analysis during the sequential finger tapping task

**Figure 4:**
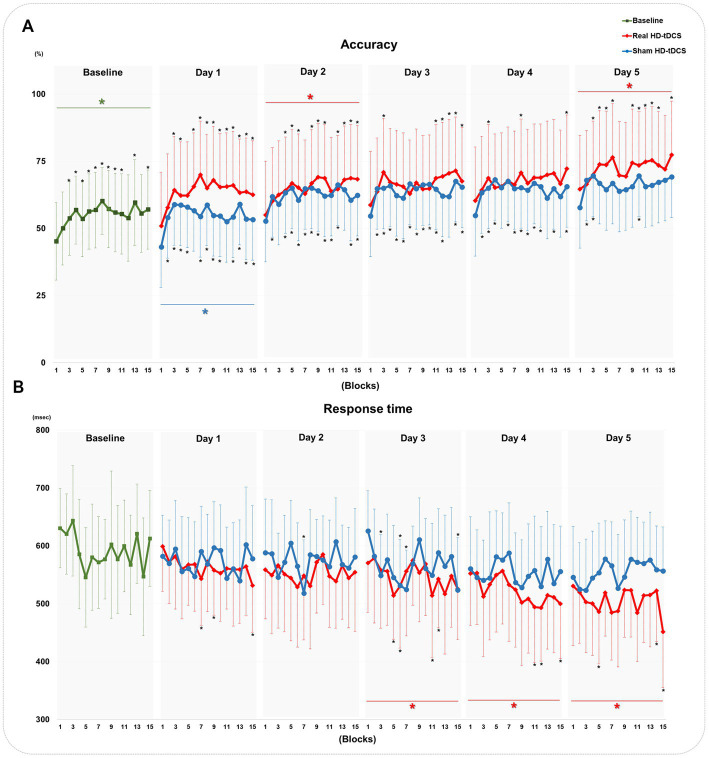
Changes in the accuracy and response time of the SFTT. **(A)** Changes in accuracy at baseline and in the real HD-tDCS and sham HD-tDCS conditions. **(B)** Changes in the response time at baseline and in the real HD-tDCS and sham HD-tDCS conditions. Green asterisks indicate statistical significance between block 1 and block 15 at baseline (Friedman test, *p* < 0.05). Red asterisks indicate statistical significance between block 1 and block 15 in the real HD-tDCS condition on each day (Friedman test, *p* < 0.05). Blue asterisks indicate statistical significance from block 1 to block 15 in the sham HD-tDCS condition on each day (Friedman test, *p* < 0.05). Black asterisks indicate statistical significance between block 1 and each other block on each measurement day (Wilcoxon signed rank test, *p < 0.05, **p < 0.01). SFTT, sequential finger tapping task; HD-tDCS, high-definition transcranial direct current stimulation.

Figure 4 shows changes in accuracy and response time during every block from baseline to day 5 in each condition. RM-ANOVA failed to demonstrate a block × condition interaction in the accuracy changes on each day. At baseline, the accuracy of the SFTT increased with statistical significance from blocks 1 to 15 (RM-ANOVA, *F* = 2.507, *p* = 0.038) (Figure 4A). In the real HD-tDCS condition, the accuracy improved significantly by block on day 2 (Friedman test, ${Χ}^{2}$ = 29.766, df = 14, *p* = 0.008) and day 5 (Friedman test, ${Χ}^{2}$ = 29.239, df = 14, *p* = 0.010) (Figure 4A). In the sham HD-tDCS condition, the accuracy tended to increase on all days; it changed significantly on block days 1, 2, and 4 (Friedman test, day 1: ${Χ}^{2}$ = 29.143, df = 14, *p* = 0.010; day 2: ${Χ}^{2}$ = 24.789, df = 14, *p* = 0.037; day 4: ${Χ}^{2}$ = 23.796, df = 14, *p* = 0.048) (Figure 4A). No significant differences in response time were found to have a block × condition interaction on any day. The response time in the SFTT tended to decrease from blocks 1 to 15 without statistical significance at baseline (Figure 4B). In the real HD-tDCS condition, the response time differed significantly by block on day 3 (Friedman test, ${Χ}^{2}$ = 34.517, df = 14, *p* = 0.002), day 4 (Friedman test, ${Χ}^{2}$ = 43.270, df = 14, *p* < 0.001), and day 5 (Friedman test, ${Χ}^{2}$ = 27.757, df = 14, *p* = 0.015). In the sham HD-tDCS condition, no statistically significant differences were observed between blocks on any day.

The SI did not show statistically significant block × condition interactions on any day. At baseline, the SI increased significantly by block (Friedman test, ${Χ}^{2}$ = 31.033, df = 14, *p* = 0.005). In the real HD-tDCS condition, the SI changed significantly by block on all days (Friedman test, day 1: ${Χ}^{2}$ = 39.487, df = 14, *p* < 0.001; day 2: ${Χ}^{2}$ = 47.534, df = 14, *p* < 0.001; day3: ${Χ}^{2}$ = 45.585, df = 14, *p* < 0.001; day 4: ${Χ}^{2}$ = 33.365, df = 14, *p* = 0.003; day 5: ${Χ}^{2}$ = 40.163, df = 14, *p* < 0.001). In the sham HD-tDCS condition, the SI changed significantly by block on day 1 (Friedman test, ${Χ}^{2}$ = 35.677, df = 14, *p* = 0.001) and day 3 (RM-ANOVA, *F* = 3.009, *p* = 0.020). The SI changes in every block from baseline to day 5 for each condition are described in Supplementary Figure 3.

## 4. Discussion

In this study, we investigated changes in cortical activation and functional connectivity during the SFTT after stroke treatment with HD-tDCS on the motor cortical area. We also examined changes in motor performance as reflected by the SFTT. Our main findings are that the HD-tDCS intervention could promote cortical activation of the ipsilesional motor area during SFTT with the affected hand. Furthermore, in the cortical network, the HD-tDCS intervention enhanced functional connectivity between M1_Ipsi_ and PM_Ipsi_. Without the application of HD-tDCS, functional connectivity between M1_Contra_ and Sn_Contra_ was promoted during motor learning after stroke. Also, the hemodynamic changes caused by the real HD-tDCS intervention were accompanied by improvement in motor performance and upper extremity function in chronic stroke patients compared with the sham HD-tDCS.

In normal motor learning, increases in cortical activation of the contralateral motor area during the early stage of motor learning and improvements in response time, rather than in accuracy, are thought to indicate successful motor learning (Park et al., 2010; Krakauer et al., 2019). This study found increases in cortical activity in the ipsilesional motor area during SFTT with the affected hand after the real HD-tDCS intervention, which was accompanied by significant improvement in SFTT response time. The *t*-values for oxyHb in the channels representing the M1_Ipsi_ during SFTT with the affected hand increased significantly by day in the real HD-tDCS condition. After the sham HD-tDCS intervention, SFTT performance did not reach the same level as with the real HD-tDCS intervention. In the normal process of motor learning, recruitment of M1 plays a key role through use-dependent mechanisms (Hardwick et al., 2013). Therefore, modulating M1 by enhancing cortical activation in stroke patients has been suggested as a strategy for improving motor learning after stroke (Lefebvre et al., 2013; Kang et al., 2016). With the focal montage provided by the 4 × 1 configuration, HD-tDCS was shown to effectively improve motor skill learning in healthy subjects (Iannone et al., 2022). Also, application of HD-tDCS to the motor area has been shown to increase task-related cortical activation of the motor area (Muthalib et al., 2014; Besson et al., 2019). Our results of enhanced cortical activation after HD-tDCS application to ipsilesional M1 with motor learning training differ from those of a previous study. Prior results showed decreased cortical activation with HD-tDCS and a simple motor task in chronic stroke patients (Kim et al., 2022). This difference could be due to the motor task paradigm. First, the duration of the motor task at 1 of our sessions was longer than that of Kim et al. (2022). Second, our motor task paradigm contains repetitions of a sequence, unlike the simple motor task of the previous study. Multiple sessions of a sequence-specific motor learning task enhance response to repetition of experience-driven changes of M1, unlike a simple motor task (Karni et al., 1998). It is conceivable that the motor task paradigm plays a critical role in the effectiveness of HD-tDCS on task-related cortical activation in chronic stroke patients. Our cortical activation results imply that HD-tDCS could augment motor performance, especially in terms of response time, by increasing cortical activation of the motor area after stroke. In other words, they suggest that HD-tDCS could alter cortical activation and motor learning patterns after stroke to better reflect the normal pattern of early-stage motor learning.

The motor cortical areas M1, PM, and SMA act as a hub for forming networks with other cortical or subcortical regions engaged in motor learning (Dahms et al., 2020). In the real HD-tDCS condition, the intensity of connection between M1_Ipsi_ and PM_Ipsi_ increased and was accompanied by a decrease in connection with M1_Contra_ and the frontal areas. With those hemodynamic changes, response time during the SFTT improved significantly. When reproducing motor sequences with precise timing, the PM plays a crucial role in temporal organization of movements by producing a rhythmic pattern of motor sequences and sending a projection to M1 to produce motor sequence outputs with optimal timing (Halsband et al., 1993). Previous findings demonstrated that performance of automatic sequential finger movements involved greater activity of the PM to compensate for reduced connections between the PM and M1 that result from degenerative changes in the brain (Wu and Hallett, 2005). After stroke, contributions of the PM that support the role of M1 represent a tract-specific structure–function relationship for improving motor performance (Schulz et al., 2012). Participants in our study had lesions including the BG, which indicate impairment in generating significant output from learned sequences to the descending motor system. Thus, strengthened functional connectivity between M1_Ipsi_ and PM_Ipsi_ after HD-tDCS indicate that PM_Ipsi_ plays an important role in supporting M1_Ipsi_ in projecting the motor output of skilled movements by inducing timing-effective motor performance of a learned skill. We also found that functional connectivity between M1_Contra_ and Sn_Contra_ was strengthened when stroke patients repeated the motor learning task without the HD-tDCS intervention. It is widely recognized that implicit sensorimotor recalibration serves to minimize motor execution errors during performance of implicit motor learning (Krakauer et al., 2019; Kim et al., 2021). In our patients, we found an effort to recruit implicit sensorimotor adaptation and thereby reduce motor execution errors during an implicit motor learning task with the affected hand that took the form of a significant strong connection between M1_Contra_ and Sn_Contra_, but not between M1_Ipsi_ and Sn_Ipsi_. The enhanced functional connectivity between M1_Contra_ and Sn_Contra_ might indicate that the sensory-motor network was strengthened in the contralesional hemisphere because of the interhemispheric imbalance after stroke (Berenguer-Rocha et al., 2020). These findings support imaging evidence from a previous study indicating that application of inhibitory brain stimulation over the contralesional sensory and motor cortex could enhance motor learning in post-stroke patients (Meehan et al., 2011). In previous findings (Mary et al., 2017), the lower resting-state connectivity between the sensorimotor cortex and other learning-related areas was related to a reduced need to perform error detection and correction in a healthy young subject. The results of the current study showed strengthened functional connectivity of M1_Ipsi_ with PM_Ipsi_, M1_Contra_, and Sn_Contra_, in chronic stroke patients with learning-related lesions. The differences in those findings imply that the strengthened functional connectivity in cortical levels induces motor learning by compensating for the role of learning-related lesions in chronic stroke patients, unlike the healthy population.

To the best of our knowledge, this is the first study to investigate the modulating effect of HD-tDCS on learning-related hemodynamic changes in chronic stroke patients with restricted subcortical lesions by analyzing changes in both cortical activation and functional connectivity at the whole brain level. Our findings provide evidence that HD-tDCS could improve motor performance during a motor learning task by increasing learning-related cortical activation in M1_Ipsi_ and strengthening the learning-related connection between M1_Ipsi_ and PM_Ipsi_. Nonetheless, this study has several limitations. First, there is a potential lack of statistical power due to our small sample size; therefore, our results cannot be generalized to the entire stroke population. Second, lack of successive recordings during repeated administrations over several weeks prevented analysis of HD-tDCS after-effects. Future research with a larger sample size in the stroke population and long-term sustainability are needed to identify the clinically relevant effects of HD-tDCS for motor learning in stroke patients. Third, we could not measure the hemodynamic changes that occurred during application of HD-tDCS. To investigate the direct mechanisms underlying HD-tDCS, future studies need to measure hemodynamic changes during HD-tDCS. Fourth, the stroke lesions of participants were diverse; most of patients had concomitant lesion of the corona radiata well as the BG. Therefore, it was not possible to interpret the results in relation only with BG lesion. To affirm the learning-related hemodynamic changes associated with specific lesions, future studies need to concentrate on stroke patients with homogenous lesions.

## 5. Conclusion

This study has demonstrated that HD-tDCS induced increases in cortical activation at M1_Ipsi_ and enhanced functional connectivity between M1_Ipsi_ and PM_Ipsi_ in chronic stroke patients. Learning-related changes in cortical activation and functional connectivity caused by HD-tDCS correlated with improved motor performance, particularly motor learning task response time. The results of our study imply that HD-tDCS to M1_Ipsi_ could allow efficient hemodynamic changes in motor network areas that promote successful motor learning among stroke patients.

## Data availability statement

The raw data supporting the conclusions of this article will be made available by the authors, without undue reservation.

## Ethics statement

The studies involving human participants were reviewed and approved by the Samsung Medical Center. The patients/participants provided their written informed consent to participate in this study. Written informed consent was obtained from the individual(s) for the publication of any potentially identifiable images or data included in this article.

## Author contributions

HK: conceptualization, methodology, formal analysis, investigation, writing—original draft, and visualization. GL: methodology and investigation. JL: conceptualization, methodology, investigation, interpretation, and writing—review and editing, supervision. Y-HK: conceptualization, resources, interpretation, writing—review and editing, supervision, project administration, and funding acquisition. All authors contributed to the article and approved the submitted version.

## Funding

This study was supported by the Korea Medical Device Development Fund grant of the Korea Government (Ministry of Science and ICT; Ministry of Trade, Industry, and Energy; Ministry of Health and Welfare; Ministry of Food and Drug Safety) (KMDF-RS-2022-00140478) and the National Research Foundation of Korea (NRF) grant funded by the Korea Government (MSIT) (no. RS-2023-00208884).

## Conflict of interest

The authors declare that the research was conducted in the absence of any commercial or financial relationships that could be construed as a potential conflict of interest.

## Publisher’s note

All claims expressed in this article are solely those of the authors and do not necessarily represent those of their affiliated organizations, or those of the publisher, the editors and the reviewers. Any product that may be evaluated in this article, or claim that may be made by its manufacturer, is not guaranteed or endorsed by the publisher.

## Footnotes

^1^ http://www.cabiatl.com/mricro/mricro/index.html

^2^ http://bisp.kaist.ac.kr/NIRS-SPM

^3^ https://sites.google.com/site/dsucore/free/optonet

## References

[ref1] Ammann C. Spampinato D. Mãrquez-Ruiz J. (2016). Modulating motor learning through transcranial direct-current stimulation: an integrative view. Front. Psychol. 7:1981. doi: 10.3389/fpsyg.2016.01981 28066300 PMC5179543

[ref2] Anwer S. Waris A. Gilani S. O. Iqbal J. Shaikh N. Pujari A. N.. (2022). Rehabilitation of upper limb motor impairment in stroke: a narrative review on the prevalence, risk factors, and economic statistics of stroke and state of the art therapies. Healthcare 10:190. doi: 10.3390/healthcare10020190 35206805 PMC8872602

[ref3] Baldwin M. L. Butler R. J. (2006). Upper extremity disorders in the workplace: costs and outcomes beyond the first return to work. J. Occup. Rehabil. 16, 296–316. doi: 10.1007/s10926-006-9043-2 16933145

[ref4] Benjamini Y. Hochberg Y. (1995). Controlling the false discovery rate: a practical and powerful approach to multiple testing. J. R. Stat. Soc. B 57, 289–300.

[ref5] Berenguer-Rocha M. Baltar A. Rocha S. Shirahige L. Brito R. Monte-Silva K. (2020). Interhemispheric asymmetry of the motor cortex excitability in stroke: relationship with sensory-motor impairment and injury chronicity. Neurol. Sci. 41, 2591–2598. doi: 10.1007/s10072-020-04350-4, PMID: 32253636

[ref6] Besson P. Muthalib M. Dray G. Rothwell J. Perrey S. (2019). Concurrent anodal transcranial direct-current stimulation and motor task to influence sensorimotor cortex activation. Brain Res. 1710, 181–187. doi: 10.1016/j.brainres.2019.01.003, PMID: 30610875

[ref7] Cuypers K. Leenus D. J. Van Den Berg F. E. Nitsche M. A. Thijs H. Wenderoth N.. (2013). Is motor learning mediated by tDCS intensity? PLoS One 8:e67344. doi: 10.1371/journal.pone.0067344, PMID: 23826272 PMC3691194

[ref8] Dahms C. Brodoehl S. Witte O. W. Klingner C. M. (2020). The importance of different learning stages for motor sequence learning after stroke. Hum. Brain Mapp. 41, 270–286. doi: 10.1002/hbm.24793, PMID: 31520506 PMC7268039

[ref9] Datta A. Bansal V. Diaz J. Patel J. Reato D. Bikson M. (2009). Gyri-precise head model of transcranial direct current stimulation: improved spatial focality using a ring electrode versus conventional rectangular pad. Brain Stimul. 2, 201–207.e1. doi: 10.1016/j.brs.2009.03.005 20648973 PMC2790295

[ref10] Delorme M. Vergotte G. Perrey S. Froger J. Laffont I. (2019). Time course of sensorimotor cortex reorganization during upper extremity task accompanying motor recovery early after stroke: an fNIRS study. Restor. Neurol. Neurosci. 37, 207–218. doi: 10.3233/RNN-180877, PMID: 31227675

[ref11] Dissanayaka T. Zoghi M. Farrell M. Egan G. F. Jaberzadeh S. (2017). Does transcranial electrical stimulation enhance corticospinal excitability of the motor cortex in healthy individuals? A systematic review and meta-analysis. Eur. J. Neurosci. 46, 1968–1990. doi: 10.1111/ejn.13640, PMID: 28699187

[ref12] Doyon J. Penhune V. Ungerleider L. G. (2003). Distinct contribution of the cortico-striatal and cortico-cerebellar systems to motor skill learning. Neuropsychologia 41, 252–262. doi: 10.1016/S0028-3932(02)00158-6, PMID: 12457751

[ref13] Esmaeilpour Z. Shereen A. D. Ghobadi-Azbari P. Datta A. Woods A. J. Ironside M.. (2020). Methodology for tDCS integration with fMRI. Hum. Brain Mapp. 41, 1950–1967. doi: 10.1002/hbm.24908, PMID: 31872943 PMC7267907

[ref14] Ferrari M. Quaresima V. (2012). A brief review on the history of human functional near-infrared spectroscopy (fNIRS) development and fields of application. Neuroimage 63, 921–935. doi: 10.1016/j.neuroimage.2012.03.049, PMID: 22510258

[ref15] Gãlvez V. Alonzo A. Martin D. Loo C. K. (2013). Transcranial direct current stimulation treatment protocols: should stimulus intensity be constant or incremental over multiple sessions? Int. J. Neuropsychopharmacol. 16, 13–21. doi: 10.1017/S1461145712000041 22310245

[ref16] Halsband U. Ito N. Tanji J. Freund H.-J. (1993). The role of premotor cortex and the supplementary motor area in the temporal control of movement in man. Brain 116, 243–266. doi: 10.1093/brain/116.1.243, PMID: 8453461

[ref17] Hamoudi M. Schambra H. M. Fritsch B. Schoechlin-Marx A. Weiller C. Cohen L. G.. (2018). Transcranial direct current stimulation enhances motor skill learning but not generalization in chronic stroke. Neurorehabil. Neural Repair 32, 295–308. doi: 10.1177/1545968318769164, PMID: 29683030 PMC6350256

[ref18] Hardwick R. M. Rottschy C. Miall R. C. Eickhoff S. B. (2013). A quantitative meta-analysis and review of motor learning in the human brain. Neuroimage 67, 283–297. doi: 10.1016/j.neuroimage.2012.11.020, PMID: 23194819 PMC3555187

[ref19] Huo C. Xu G. Li W. Xie H. Zhang T. Liu Y.. (2021). A review on functional near-infrared spectroscopy and application in stroke rehabilitation. Med. Nov. Technol. Dev. 11:100064. doi: 10.1016/j.medntd.2021.100064

[ref20] Iannone A. Santiago I. Ajao S. T. Brasil-Neto J. Rothwell J. C. Spampinato D. A. (2022). Comparing the effects of focal and conventional tDCS on motor skill learning: a proof of principle study. Neurosci. Res. 178, 83–86. doi: 10.1016/j.neures.2022.01.006, PMID: 35123828 PMC9042790

[ref21] Kami A. Meyer G. Jezzard P. Adams M. M. Turner R. Ungerleider L. G. (1995). Functional MRI evidence for adult motor cortex plasticity during motor skill learning. Nature 377, 155–158. doi: 10.1038/377155a0 7675082

[ref22] Kang N. Summers J. J. Cauraugh J. H. (2016). Transcranial direct current stimulation facilitates motor learning post-stroke: a systematic review and meta-analysis. J. Neurol. Neurosurg. Psychiatry 87, 345–355. doi: 10.1136/jnnp-2015-311242, PMID: 26319437

[ref23] Karni A. Meyer G. Rey-Hipolito C. Jezzard P. Adams M. M. Turner R.. (1998). The acquisition of skilled motor performance: fast and slow experience-driven changes in primary motor cortex. Proc. Natl. Acad. Sci. U. S. A. 95, 861–868. doi: 10.1073/pnas.95.3.861, PMID: 9448252 PMC33809

[ref24] Kim H. E. Avraham G. Ivry R. B. (2021). The psychology of reaching: action selection, movement implementation, and sensorimotor learning. Annu. Rev. Psychol. 72, 61–95. doi: 10.1146/annurev-psych-010419-051053, PMID: 32976728 PMC8514106

[ref25] Kim H. Kim J. Lee G. Lee J. Kim Y.-H. (2022). Task-related hemodynamic changes induced by high-definition Transcranial direct current stimulation in chronic stroke patients: an uncontrolled pilot fNIRS study. Brain Sci. 12:453. doi: 10.3390/brainsci12040453, PMID: 35447985 PMC9028267

[ref26] Kim Y.-H. You S. H. Ko M.-H. Park J.-W. Lee K. H. Jang S. H.. (2006). Repetitive transcranial magnetic stimulation–induced corticomotor excitability and associated motor skill acquisition in chronic stroke. Stroke 37, 1471–1476. doi: 10.1161/01.STR.0000221233.55497.51, PMID: 16675743

[ref27] Krakauer J. W. Hadjiosif A. M. Xu J. Wong A. L. Haith A. M. (2019). Motor learning. Compr. Physiol. 9, 613–663. doi: 10.1002/cphy.c170043, PMID: 30873583

[ref28] Kuo H.-I. Bikson M. Datta A. Minhas P. Paulus W. Kuo M.-F.. (2013). Comparing cortical plasticity induced by conventional and high-definition 4× 1 ring tDCS: a neurophysiological study. Brain Stimul. 6, 644–648. doi: 10.1016/j.brs.2012.09.010, PMID: 23149292

[ref29] Lachaux J. P. Rodriguez E. Martinerie J. Varela F. J. (1999). Measuring phase synchrony in brain signals. Hum. Brain Mapp. 8, 194–208. doi: 10.1002/(SICI)1097-0193(1999)8:4<194::AID-HBM4>3.0.CO;2-C 10619414 PMC6873296

[ref30] Langhorne P. Coupar F. Pollock A. (2009). Motor recovery after stroke: a systematic review. Lancet Neurol. 8, 741–754. doi: 10.1016/S1474-4422(09)70150-4 19608100

[ref31] Lee G. Park J.-S. Jung Y.-J. (2019). OptoNet: a MATLAB-based toolbox for cortical network analyses using functional near-infrared spectroscopy. Opt. Eng. 59:061602. doi: 10.1117/1.OE.59.6.061602

[ref32] Lee G. Park J.-S. Lee J. Kim J. Jung Y.-J. Kim Y.-H. (2020). OptoNet II: an advanced MATLAB-based toolbox for functional cortical connectivity analysis with surrogate tests using fNIRS. IEEE Access 9, 15983–15991. doi: 10.1109/ACCESS.2020.3042808

[ref33] Lee S.-H. Lee H.-J. Shim Y. Chang W. H. Choi B.-O. Ryu G.-H.. (2020). Wearable hip-assist robot modulates cortical activation during gait in stroke patients: a functional near-infrared spectroscopy study. J. Neuroeng. Rehabil. 17, 1–8. doi: 10.1186/s12984-020-00777-0 33121535 PMC7596937

[ref34] Lefebvre S. Jann K. Schmiesing A. Ito K. Jog M. Schweighofer N.. (2019). Differences in high-definition transcranial direct current stimulation over the motor hotspot versus the premotor cortex on motor network excitability. Sci. Rep. 9, 1–15. doi: 10.1038/s41598-019-53985-7 31772347 PMC6879500

[ref35] Lefebvre S. Laloux P. Peeters A. Desfontaines P. Jamart J. Vandermeeren Y. (2013). Dual-tDCS enhances online motor skill learning and long-term retention in chronic stroke patients. Front. Hum. Neurosci. 6:343. doi: 10.3389/fnhum.2012.00343 23316151 PMC3541043

[ref36] Leff D. R. Orihuela-Espina F. Elwell C. E. Athanasiou T. Delpy D. T. Darzi A. W.. (2011). Assessment of the cerebral cortex during motor task behaviours in adults: a systematic review of functional near infrared spectroscopy (fNIRS) studies. Neuroimage 54, 2922–2936. doi: 10.1016/j.neuroimage.2010.10.058, PMID: 21029781

[ref37] Lewis P. A. Miall R. C. (2003). Distinct systems for automatic and cognitively controlled time measurement: evidence from neuroimaging. Curr. Opin. Neurobiol. 13, 250–255. doi: 10.1016/S0959-4388(03)00036-9, PMID: 12744981

[ref38] Martïnez-Përez V. Campoy G. Palmero L. B. Fuentes L. J. (2020). Examining the dorsolateral and ventromedial prefrontal cortex involvement in the self-attention network: a randomized, sham-controlled, parallel group, double-blind, and multichannel HD-tDCS study. Front. Neurosci. 14:683. doi: 10.3389/fnins.2020.00683, PMID: 32760241 PMC7371986

[ref39] Mary A. Wens V. Op De Beeck M. Leproult R. De Tiêge X. Peigneux P. (2017). Resting-state functional connectivity is an age-dependent predictor of motor learning abilities. Cereb. Cortex 27, 4923–4932. doi: 10.1093/cercor/bhw286, PMID: 27655931

[ref40] Meehan S. K. Dao E. Linsdell M. A. Boyd L. A. (2011). Continuous theta burst stimulation over the contralesional sensory and motor cortex enhances motor learning post-stroke. Neurosci. Lett. 500, 26–30. doi: 10.1016/j.neulet.2011.05.237, PMID: 21683125

[ref41] Muller C. O. Muthalib M. Mottet D. Perrey S. Dray G. Delorme M.. (2021). Recovering arm function in chronic stroke patients using combined anodal HD-tDCS and virtual reality therapy (ReArm): a study protocol for a randomized controlled trial. Trials 22, 1–18. doi: 10.1186/s13063-021-05689-5 34702317 PMC8549202

[ref42] Muthalib M. Dutta A. Besson P. Rothwell J. Ward T. Perrey S. Comparison of online vs offline effects of HD-tDCS induced modulation of cortical sensorimotor networks using a combined fNIRS-EEG setup. Poster Presented at the International Society on Oxygen Transport to Tissues (ISOTT) Conference. London (2014).

[ref43] Park J.-W. Kim Y.-H. Jang S. H. Chang W. H. Park C.-H. Kim S. T. (2010). Dynamic changes in the cortico-subcortical network during early motor learning. Neurorehabilitation 26, 95–103. doi: 10.3233/NRE-2010-0540, PMID: 20203374

[ref44] Russo C. Souza Carneiro M. I. Bolognini N. Fregni F. (2017). Safety review of transcranial direct current stimulation in stroke. Neuromodulation 20, 215–222. doi: 10.1111/ner.12574 28220641 PMC5389927

[ref45] Schulz R. Park C.-H. Boudrias M.-H. Gerloff C. Hummel F. C. Ward N. S. (2012). Assessing the integrity of corticospinal pathways from primary and secondary cortical motor areas after stroke. Stroke 43, 2248–2251. doi: 10.1161/STROKEAHA.112.662619 22764214 PMC3477824

[ref46] Shoushtarian M. Alizadehsani R. Khosravi A. Acevedo N. Mckay C. M. Nahavandi S.. (2020). Objective measurement of tinnitus using functional near-infrared spectroscopy and machine learning. PLoS One 15:e0241695. doi: 10.1371/journal.pone.0241695, PMID: 33206675 PMC7673524

[ref47] Spampinato D. Celnik P. (2021). Multiple motor learning processes in humans: defining their neurophysiological bases. Neuroscientist 27, 246–267. doi: 10.1177/1073858420939552, PMID: 32713291 PMC8151555

[ref48] Sveen U. Bautz-Holter E. Margrethe Sodring K. Bruun Wyller T. Laake K. (1999). Association between impairments, self-care ability and social activities 1 year after stroke. Disabil. Rehabil. 21, 372–377. 10503978 10.1080/096382899297477

[ref49] Tak S. Jang K. E. Jung J. Jang J. Jeong Y. Ye J. C. NIRS-SPM: Statistical parametric mapping for near infrared spectroscopy. Multimodal Biomedical Imaging III. San Jose, California, United States: International Society for Optics and Photonics (2008).

[ref50] Thair H. Holloway A. L. Newport R. Smith A. D. (2017). Transcranial direct current stimulation (tDCS): a beginner’s guide for design and implementation. Front. Neurosci. 11:641. doi: 10.3389/fnins.2017.00641, PMID: 29213226 PMC5702643

[ref51] Villamar M. F. Volz M. S. Bikson M. Datta A. Dasilva A. F. Fregni F. (2013). Technique and considerations in the use of 4x1 ring high-definition transcranial direct current stimulation (HD-tDCS). J. Vis. Exp. 77:e50309. doi: 10.3791/50309-v PMC3735368 23893039

[ref52] Wu T. Hallett M. (2005). The influence of normal human ageing on automatic movements. J. Physiol. 562, 605–615. doi: 10.1113/jphysiol.2004.076042, PMID: 15513939 PMC1665504

[ref53] Yaqub M. A. Woo S.-W. Hong K.-S. (2018). Effects of HD-tDCS on resting-state functional connectivity in the prefrontal cortex: an fNIRS study. Complexity 2018, 1–13. doi: 10.1155/2018/1613402

[ref54] Ye J. C. Tak S. Jang K. E. Jung J. Jang J. (2009). NIRS-SPM: statistical parametric mapping for near-infrared spectroscopy. Neuroimage 44, 428–447. doi: 10.1016/j.neuroimage.2008.08.036 18848897

[ref55] Zimeo Morais G. A. Balardin J. B. Sato J. R. (2018). fNIRS Optodes’ location decider (fOLD): a toolbox for probe arrangement guided by brain regions-of-interest. Sci. Rep. 8, 1–11. doi: 10.1038/s41598-018-21716-z 29463928 PMC5820343

