# Supplementary material for: Alterations in learning-related cortical activation and functional connectivity by high-definition transcranial direct current stimulation after stroke: an fNIRS study
Source: Front Neurosci. 2023 Jun 2;17:1189420. doi: 10.3389/fnins.2023.1189420 (PMC10275383; doi:10.3389/fnins.2023.1189420)

Supplementary Figures

Supplementary Figure 1. Lesion map. Right lesions are flipped to the left hemisphere, and all lesions are overlaid on the left hemisphere. The colored bar indicates the number of participants.

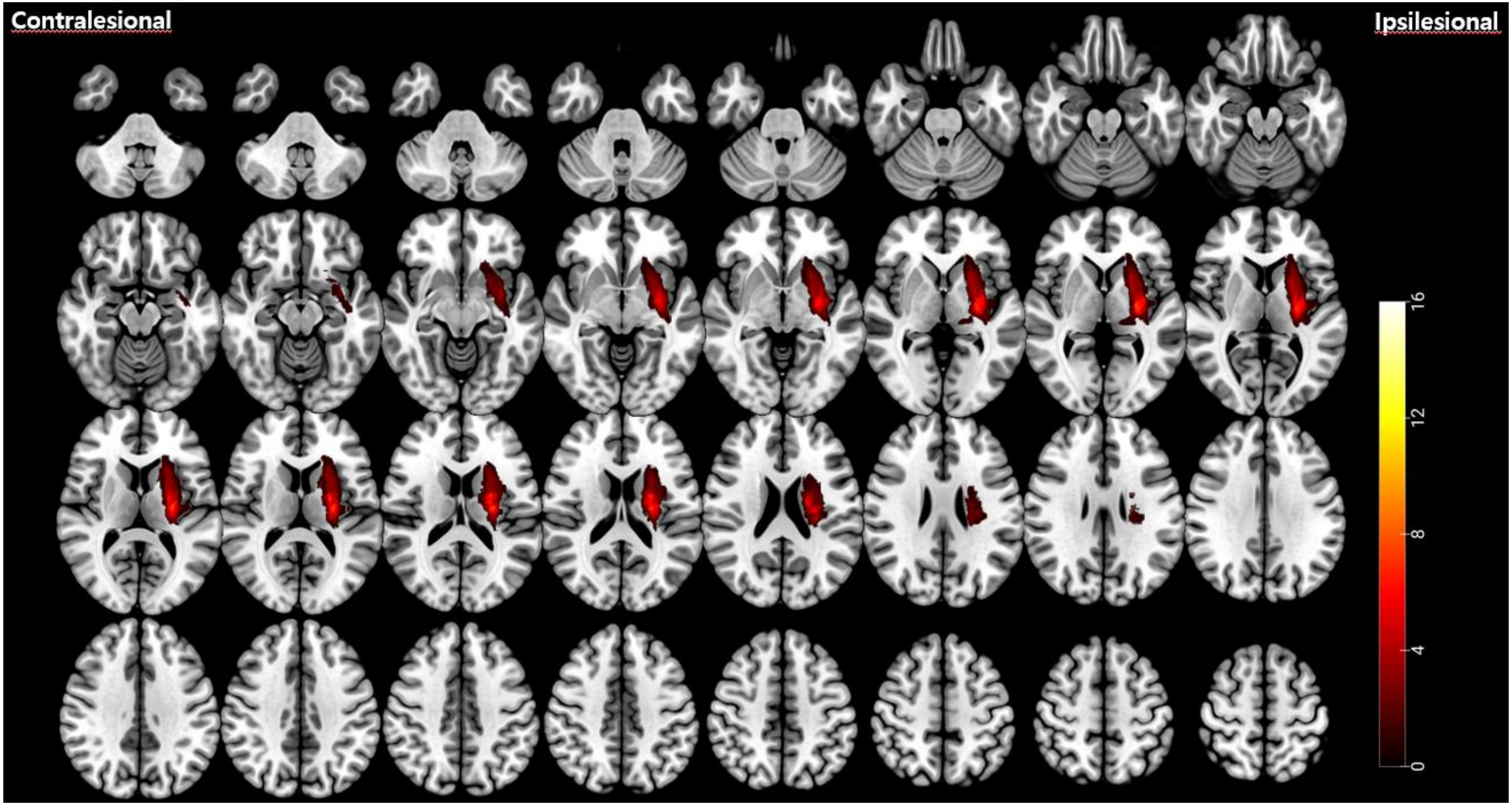

Supplementary Figure 2. Changes in functional connectivity between the contralesional M1 and other ROIs during the SFTT with the affected hand. The letters in green circles indicate the names of the ROIs, and the colored lines represent functional connectivity between the contralesional M1 and each ROI. The numbers above each green circle indicate the mean PLV between the contralesional M1 and that ROI. The functional connectivity line is presented as a warmer color if the PLV was close to 1 and a cooler color if the PLV was close to 0, and only high-value lines (threshold > 0.2) are presented. The PLV between the contralesional M1 and sensory cortex increased on day 3, day 4 and day 5 compared with baseline in the sham HD-tDCS condition (Wilcoxon signed rank test, $p < 0.05$). MPF, medial prefrontal cortex; Fr, frontal area; M1, primary motor cortex; SMA, supplementary motor area; PM, premotor cortex; Sn, sensory cortex; Pr, parietal cortex; Tm, temporal lobe; Occ, occipital lobe

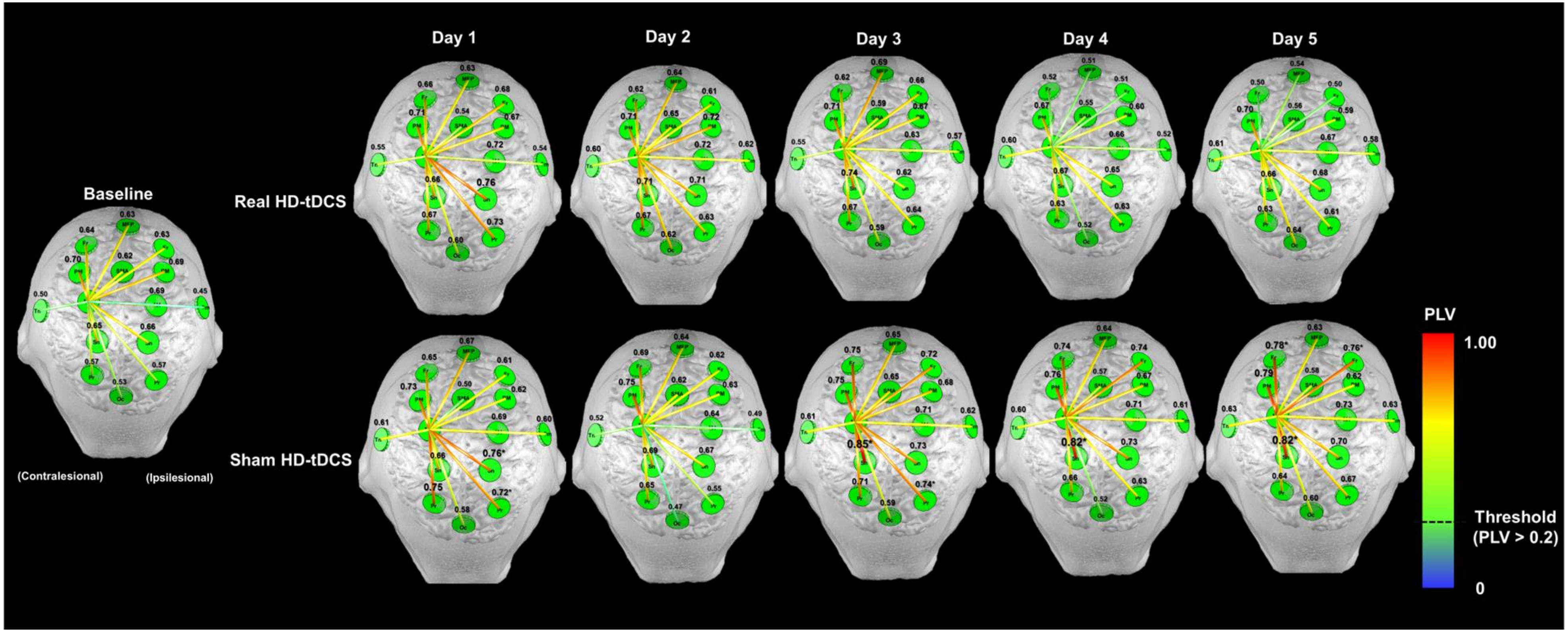

Supplementary Figure 3. Changes in the skill index of the SFTT. Red asterisks indicate statistical significance between block 1 and block 15 in the real HD-tDCS condition on each day (Friedman test, *$p < 0.05$, ** $p < 0.01$). Blue asterisks indicate statistical significance between block 1 and block 15 in the sham HD-tDCS condition on each day (Friedman test or RM-ANOVA, *$p < 0.05$, ** $p < 0.01$). Black asterisks indicate statistical significance between block 1 and each other block on each measurement day (Wilcoxon signed rank test, $p < 0.05$). SFTT, sequential finger tapping task; HD-tDCS, high-definition transcranial direct current stimulation

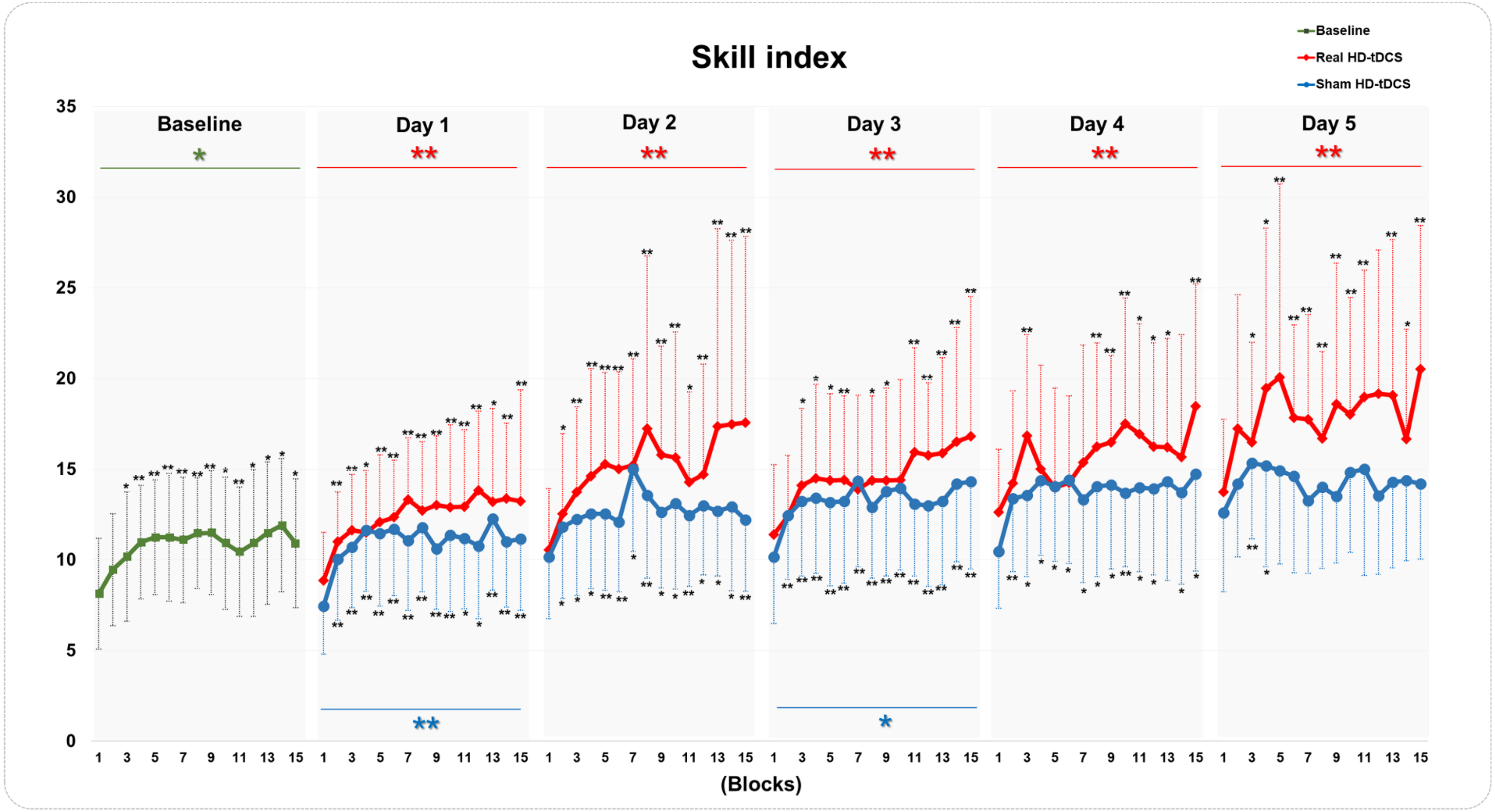

Supplement: Supplementary file 1 [file Data_Sheet_1.PDF]
